# Population status, habitat preferences and predictive current and future distributions of three endangered *Silene* species under changing climate

**DOI:** 10.3389/fpls.2024.1336911

**Published:** 2024-06-20

**Authors:** Mohamed Abdelaal, Arwa Abdulkreem AL-Huqail, Suliman Mohammed Suliman Alghanem, Haifa Abdulaziz Sakit Alhaithloul, Sami Asir Al-Robai, Amany H. A. Abeed, Mohammed A. Dakhil, Reham F. El-Barougy, Aya A. Yahia

**Affiliations:** ^1^ Department of Botany, Faculty of Science, Mansoura University, Mansoura, Egypt; ^2^ Department of Biology, College of Science, Princess Nourah bint Abdulrahman University, Riyadh, Saudi Arabia; ^3^ Department of Biology, College of Science, Qassim University, Buraydah, Saudi Arabia; ^4^ Biology Department, College of Science, Jouf University, Sakaka, Saudi Arabia; ^5^ Department of Biology, Faculty of Science, Al-Baha University, Al-Baha, Saudi Arabia; ^6^ Department of Botany and Microbiology, Faculty of Science, Assiut University, Assiut, Egypt; ^7^ Botany and Microbiology Department, Faculty of Science, Helwan University, Cairo, Egypt; ^8^ School of Ecology and Environment, Northwestern Polytechnical University, Xi’an, China; ^9^ Botany and Microbiology Department, Faculty of Science, Damietta University, Damietta, Egypt

**Keywords:** arid region, conservation, Egypt, ensemble model, global warming

## Abstract

One of the most crucial steps in the practical conservation of endangered endemic mountain plants is to address their population size status and habitat requirements concurrently with understanding their response to future global warming. Three endangered *Silene* species—*Silene leucophylla* Boiss., *S. schimperiana* Boiss., and *S. oreosinaica* Chowdhuri—in Egypt were the focus of the current study. These species were examined for population status change, habitat quality variables (topography, soil features, and threats), and predictive current and future distributions. To find population size changes, recent field surveys and historical records were compared. Using Random Forest (RF) and Canonical Correspondence Analysis (CCA), habitat preferences were assessed. To forecast present-day distribution and climate change response, an ensemble model was used. The results reported a continuous decline in the population size of the three species. Both RF and CCA addressed that elevation, soil texture (silt, sand, and clay fractions), soil moisture, habitat-type, chlorides, electric conductivity, and slope were among the important variables associated with habitat quality. The central northern sector of the Saint Catherine area is the hotspot location for the predictive current distribution of three species with suitable areas of 291.40, 293.10, and 58.29 km^2^ for *S. leucophylla, S. schimperiana,* and *S. oreosinaica,* respectively. Precipitation-related variables and elevation were the key predictors for the current distribution of three *Silene* species. In response to climate change scenarios, the three *Silene* species exhibited a gradual contraction in the predictive suitable areas with upward shifts by 2050 and 2070. The protection of these species and reintroduction to the predicted current and future climatically suitable areas are urgent priorities. Ex-situ conservation and raised surveillance, as well as fenced enclosures may catapult as promising and effective approaches to conserving such threatened species.

## 1 Introduction

Climate change, land use, and over-exploitation all contribute to global biodiversity loss (Dawson et al., 2011; Cahill et al., 2013; Dagnino et al., 2020). Recognizing the influence of environmental factors on the regional distribution of plants is one of the main goals of conservation biology. The climate is among the environmental factors that affect plants’ habitat, distribution, ecology, and phenology (Hu et al., 2015; Liu et al., 2021). Climate change will continue to have an apparent impact on living organisms and ecosystems at all levels of ecological organization: shifts in geographic range, population, and life-history changes, changes in the structure and functioning of ecosystems, and changes in the species composition of communities (McCarty, 2001). Therefore, one of the most important challenges in conserving biodiversity is to anticipate how to face the expected climate change and search for precautionary measures to reduce its effect on species and communities (Walther et al., 2002; Root et al., 2003; Thuiller, 2004).

As a result of climate change, some plants may expand or contract their geographic range, while others may shift their range. In particular, endemic plant species have narrow ecological niches and poor dispersal abilities within well-defined habitats. Therefore, at global, national, or even local scales, endemic species are included in the prioritization of conservation efforts (Orsenigo et al., 2018). Endemic montane plants may not be qualified to adapt and maintain pace with new climate conditions and consequently, might migrate/shift to more favorable habitats or become more susceptible to habitat loss (Engler et al., 2009; Dullinger et al., 2012; Cuena-Lombraña et al., 2018; Hoorn et al., 2018; Abdelaal et al., 2019). Moreover, endemic species with isolated populations at mountain barriers and seed dispersal limitations are more vulnerable to extinction risk (Trew and Maclean, 2021). Hence, identifying and understanding the potential effects of upcoming climate change scenarios on species distribution ranges, particularly, endemic plant species, are critical steps in conserving and restoring these species (Behroozian et al., 2020; Dhyani et al., 2021).

The Saint Catherine Protectorate (St. Catherine) is located within the Sinai Peninsula in the Sinaico-Arabian biogeographic sector and Sinaic subsector of Egypt (Abdelaal et al., 2020b). It is considered Egypt’s most floristically rich territory, hosting 472 vascular plant species, including 14 exclusive endemics (Fayed et al., 2004; Mosallam, 2007; Abdelaal et al., 2018). Such phytodiversity is driven by the presence of different microhabitats such as wadi beds, terraces, rocky cliffs, slopes, gorges, caves, and basins (Shaltout et al., 2015) with an elevation gradient up to ca. 2641 m above sea level (a.s.l.). For Egypt (including St. Catherine), during a few decades (1971–2000), the average annual temperature rose by 0.62°C, which exceeded the typical rise worldwide of 0.17°C (Domroes and El-Tantawi, 2005; IPCC, 2014). Therefore, Egypt is an arid country vulnerable to the effects of climate change in particular.

The genus *Silene* L. (Family Caryophyllaceae) consists of ca. 850–900 species distributed in the Mediterranean region and Asia (Oxelman et al., 2000; Jafari et al., 2020; Martín-Gómez et al., 2020). In the flora of Egypt, *Silene* is represented by 29 species, three of which (*Silene leucophylla* Boiss., *S. schimperiana* Boiss., and *S. oreosinaica* Chowdhuri) are exclusive endemics to the St. Catherine Protectorate (Boulos, 2009). These three endemics are the focus of the current study.

*Silene leucophylla* is a perennial hemicryptophyte, up to 40 cm tall, with obovate entire rosette leaves (Boulos, 2009). It is restricted to rocky cliffs within mountains. *Silene leucophylla* is used as a grazing plant and in traditional medicine to treat diarrhea, leprosy, and inflammation (Mosallam, 2007). It is categorized as an endangered plant (Saker et al., 2011). *Silene schimperiana* is a perennial hemicryptophyte with stems 50–80 cm long and narrow linear-spathulate rosette leaves, confined to rocky crevices of mountains (Boulos, 2009). It is restricted to granite rocky soils within mountains. It is also assessed as an endangered plant (Mosallam, 2007). *Silene oreosinaica* is a perennial caespitose chamaephyte, 20–35 cm tall, with oblong-lanceolate rosette leaves. It is restricted to St. Catherine and assessed as a critically endangered plant (Radford et al., 2011).

Species Distribution Models (SDMs) have been established as one of the most commonly used tools in conservation ecology (Guisan and Thuiller, 2005). By correlating species occurrence data with environmental factors, SDMs can predict the potential distribution ranges of species under current and future conditions (Guisan and Zimmermann, 2000), help to identify the most contributing environmental factors and assist in recognizing suitable sites for restoration or translocation of endemic and rare species (Mccain and Colwell, 2011; Fois et al., 2015, Fois et al., 2018; Abdelaal et al., 2019; Wang et al., 2019; Abdelaal et al., 2020a). Occasionally, ecological data are complex, unbalanced, and contain missing values. Therefore, as being SDMs limitations, modeling techniques might originate non-significant predictions, leaving high amounts of unexplained variation leading to underfit models, having insufficient suppleness to describe observed occurrence–environment relationships, and originating a misinterpretation of the factors shaping species distributions (Dutra Silva et al., 2017).

To the best of our knowledge, no earlier research has addressed the population status, habitat preferences, and potential predictive current and future distributions of the three target *Silene* species. In this context, the objectives of this study were to: a) detect the changes in population size of three *Silene* species as compared with previous historical data, b) determine the habitat preferences for study *Silene* species; c) model the potential current distribution and key environmental variables; and d) identify the potential geographic changes in response to climate change.

## 2 Materials and methods

### 2.1 Study site, occurrence records and population status of three *Silene* species

**Figure 1:**
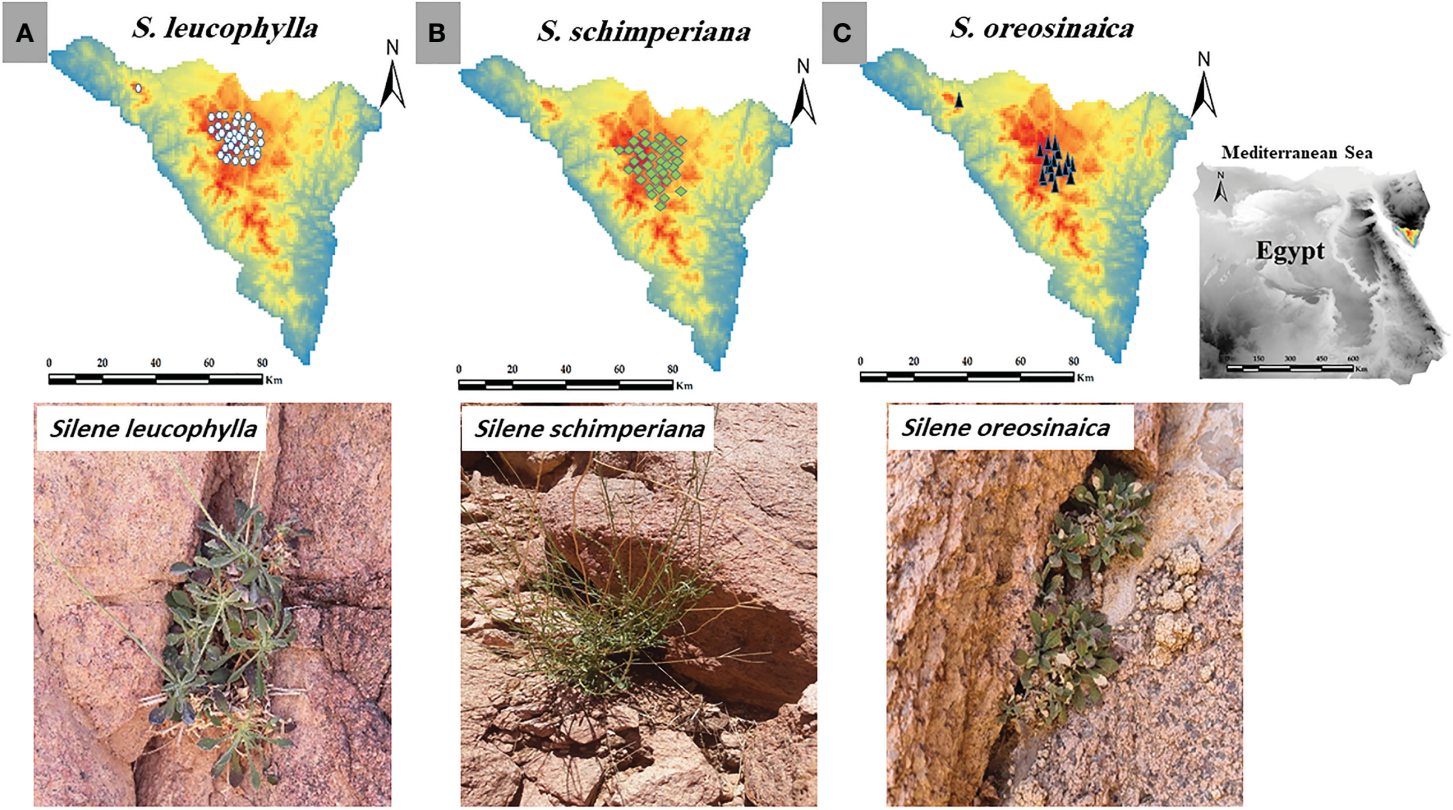
Study area, current occurrence records and morphology of **(A)** *Silene leucophylla*, **(B)** *S. schimperiana*, and **(C)** *S. oreosinaica*.

The present study was conducted in the Saint Catherine Protectorate in the Sinaic biogeographic subsector of Egypt (**Figure 1**). St. Catherine is an igneous massif with an approximate surface area of 5196 km^2^ and an elevation of up to 2641 m a.s.l. Due to its location, high mountains, and arid to hyper-arid climates, St. Catherine is characterized by unique habitats, biodiversity, endemism, landforms, and geologic structure (Moustafa and Klopatek, 1995; Moustafa et al., 2001). The mean annual rainfall (1970–2020) reaches 50.20 mm during the winter. However, during the years 2014 (Moustafa et al., 2017) and 2020 (personal observation), unpredictable one-day floods with snowfall on the mountain tops have been recorded. The average annual temperature fluctuated from 8.6°C in January to 25.5°C in August. The foremost threats in St. Catherine include drought, overgrazing, overcollection, and tourist activities.

After consultation with literature, websites, and local and international herbaria, the georeferenced occurrence records of all populations of *S. leucophylla*, *S. schimperiana*, and *S. oreosinaica* were collected from direct field surveys (**Figure 1**). *Silene leucophylla*, *S. schimperiana*, and *S. oreosinaica* were recorded in 8, 6, and 3 sites, respectively. For each *Silene* species, the biannual historic (2016–2020) population data (total number of individuals) were obtained from previous databases and reports of the last five years (unpublished data), while the current (2021–2022) population size was directly obtained from field observations. In addition, the importance value index (IVI, out of 200) was evaluated for each species by sum of both relative density and relative cover within several permanent plots of 25 m^2^ each (Kent, 2011).

### 2.2 Habitat preferences for three *Silene* species

In each site, elevation, slope, and aspect were recorded or calculated. For soil analysis, bulk soil samples were collected, sieved, and air-dried. Soil physical (texture and moisture content) and chemical features (pH, electric conductivity, organic matter, calcium carbonates, chlorides, sulphates, potassium, calcium, and magnesium) were estimated. pH and electric conductivity(EC) were measured in 1:5 w/v soil solutions by pH meter (Lutron pH 206) and conductivity meter (Corning, NY, USA), respectively. Organic matter (OM) was estimated by ignition in a muffle furnace at 450°C for 4 h. Soil analyses were carried out according to Burt (2004). Microhabitat type was recorded and ranked as follows: (1): slopes, (2): gorges, and (3): terraces. Field observations on threat type sign was ranked as follow: (1): drought, (2): over-grazing, and (3): over-collection, and distance to the nearest track/road were also documented for each *Silene* species in all localities. All the commonly associated species in the surveyed plots with the three *Silene* species were recorded. All variables were tested against normality and transformed when necessary. To find the most important variable for the habitat preference of three *Silene* species, the “*randomForest*” (RF) package (Breiman, 2001) in R software was used. In addition, Canonical Correspondence Analysis (CCA) in the “*vegan*” package was used to address the relationship between measured environmental variables and the abundance of three *Silene* species. One–way ANOVA was applied to test the significance of habitat quality variables.

### 2.3 Predictive current and future distributions using ensemble modelling

To avoid overfitting bias, the occurrence records of each *Silene* species were filtered by selecting a random point within a single raster cell of 1 km × 1 km in ArcGIS 10.7.1 (Fois et al., 2018; Smeraldo et al., 2018; Abdelaal et al., 2019). To create predictive models, 90, 75, and 40 occurrence records for *S. leucophylla*, *S. schimperiana*, and *S. oreosinaica*, respectively, were used. For the current period (1970–2000), 19 bioclimatic variables (BIO1–BIO19) were downloaded from the WorldClim v.2.1 portal at a resolution of ~1 km^2^ (http://www.worldclim.org) (Fick and Hijmans, 2017) (**Supplementary Table S1**). In addition, topographical factors (elevation, aspect, and slope) were retrieved through the Shuttle Radar Topographic Mission (SRTM) from EarthEnv at 1 km^2^ resolution (https://www.earthenv.org/topography) (Amatulli et al., 2018).

On the other hand, to avoid highly correlated variables, a Variance Inflation Factor (VIF) with a threshold of VIF< 5 was applied using the ‘*usdm’* package (Naimi, 2017) in the R-environment (R Core Team, 2022). In the end, 12 variables: temperature seasonality (BIO4); the maximum temperature of the warmest month (BIO5), mean temperature of the wettest quarter (BIO8), mean temperature of the warmest quarter (BIO10), mean temperature of the coldest quarter (BIO11), precipitation of the driest month (BIO14), precipitation of the wettest month, precipitation seasonality (BIO15), precipitation of the driest quarter (BIO17), precipitation of the warmest quarter (BIO18), precipitation of the coldest quarter (BIO19), elevation and aspect were kept for the modelling process (**Table 1**).

**Table 1:** List of 12 environmental predictors used to model the potentially suitable areas for the studied *Silene* species with their Variance Inflation Factors (VIF< 5).

Environmental variable	Code and unit	VIF
Temperature-related variables		
Temperature seasonality (SD × 100)	BIO4	4.88
Mean temperature of wettest quarter	BIO8 (°C)	4.05
Mean temperature of coldest quarter	BIO11 (°C)	4.34
Mean temperature of warmest quarter	BIO10 (°C)	4.15
Max temperature of warmest month	BIO5 (°C)	3.15
Precipitation-related variables		
Precipitation of driest month	BIO14 (mm)	3.53
Precipitation of driest quarter	BIO17 (mm)	2.59
Precipitation seasonality	BIO15	4.00
Precipitation of coldest quarter	BIO19 (mm)	2.76
Precipitation of warmest quarter	BIO18 (mm)	4.85
Topographic variables		
Elevation	Elev (m. a.s.l.)	4.10
Aspect	Aspect (°)	4.98

For future climate projections, the ensemble average of the outputs of CCSM4 and HadGEM2-CC global climate models (GCMs) were used to represent the future potential distributions of the three *Silene* species in Egypt. In addition, two Representative Concentration Pathways (RCPs): optimistic RCP2.6 and pessimistic RCP8.5, for two periods: 2050 and 2070 were downloaded from WorldClim v.2 (http://www.worldclim.org) at ~1 km^2^ resolution. The RCP2.6 scenario represents a low forcing level of 3 W m^-2^ maximum, whereas the RCP8.5 scenario depicts a rising radiative forcing level of 8.5 W m^-2^ by 2100 (van Vuuren et al., 2011).

To reduce bias and uncertainties in model prediction, an ensemble model of three algorithms was constructed (Thuiller et al., 2019) to predict the current and future distributions of *Silene* species. The three algorithms included GLM (Generalized Linear Model), RF (Random Forest), and BRT (Boosting Regression Trees). The GLM is a regression model, while RF and BRT are machine learning methods. The three selected models (GLM, RF and BRT) have excellent stability and transferability than other models (El-Barougy et al., 2023). Using the R *sdm* package (Naimi and Araújo, 2016), 70% training data, and 30% testing data, the species distribution models were conducted. The maximum training sensitivity plus specificity (MTSS) threshold was applied, which is an excellent selection method to avoid over-prediction and under-prediction errors (Liu et al., 2016; Guisan et al., 2017). To assess the models’ performance, two metrics were considered: Area Under the Curve (AUC) and True Skill Statistics (TSS) (Allouche et al., 2006; Guisan et al., 2017). The TSS and AUC values are not affected by the prevalence of the species occurrence points or the size of the study region. Higher values of both metrics (close to 1) imply high models’ accuracy and a strong correlation between model prediction and species’ real distributions. Depending on the MTSS values and by using the reclassify tool in ArcGIS, the probability maps under current and future conditions for each *Silene* species were categorized into four suitability classes as follows: unsuitable, low, moderate, and high. To evaluate the spatial range changes in the predicted distributions of the studied species under current and future climate scenarios (RCP2.6 and RCP8.5), two suitability classes were distinguished after applying the MTSS threshold: unsuitable and suitable areas. The predictive models were compared, and changes in suitable areas were calculated.

## 3 Results

### 3.1 Population status change of three *Silene* species

**Figure 2:**
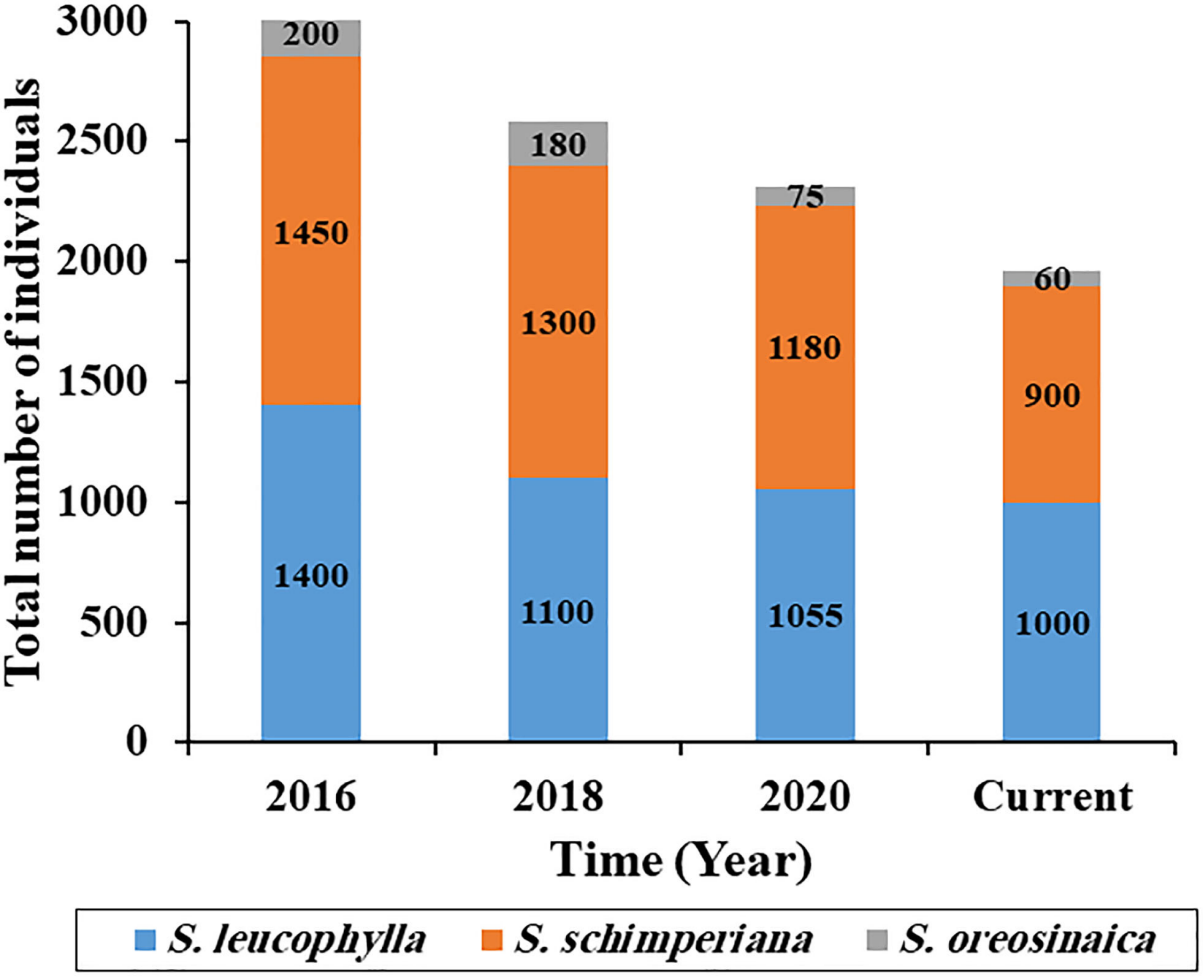
Population size change of three *Silene* species in the last five years.

As compared with the last five years, there is a gradual loss in the population size of three *Silene* species (**Figure 2**). For *S. leucophylla*, the current population size is 1000 individuals with loss percentages of 28.6, 9.1, and 5.2% when compared with the years 2016, 2018, and 2020, respectively. The current population sizes for *S. schimperiana* and *S. oreosinaica* are 900 and 60 individuals, respectively.

According to recent field observations (2021–2022), the population and microhabitat features for three *Silene* species were recorded (**Supplementary Table S2**). The population features varied among *Silene* species. For *S. leucophylla*, the total number of individuals is 1000 individuals with 69 mature individuals, and distributed in eight sites in St. Catherine. *Silene leucophylla* survives at an elevation extent between 1700 and 2200 m a.s.l. and grows in terraces, slopes, and gorges microhabitats in the rocky crevices of mountains in all aspect directions except south and flat. On the other hand, the current population size of *S. schimperiana* is 900 individuals with 51 mature individuals, spread across six sites in St. Catherine. It grows in rocky crevices at an elevation range of 1300–2300 m inside terraces, gorges, and slopes microhabitats. *Silene oreosinaica* had the smallest population size of 60 individuals, including 28 matures, and was found only in three sites on slopes microhabitats with a north-facing aspect and a narrow elevation range of 2000 to 2300 m.

Additionally, from our field observations, the populations of all target *Silene* species were sporadically distributed and severely fragmented by mountain barriers, with a continuous decline in the quality of their habitats and population size. The main threats to the three species were overgrazing by feral donkeys and domestic animals, drought, and overcollection for scientific research. Except for *S. schimperiana*, there are no fenced enclosures established for long-term monitoring of *S. leucophylla* and *S. oreosinaica*. The other microhabitat features and commonly associated species are displayed in **Supplementary Table S2**.

### 3.2 Habitat preferences for *Silene* species

**Figure 3:**
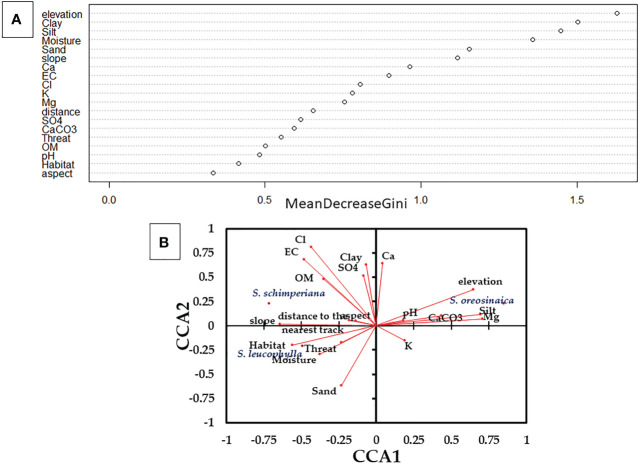
Variables importance that affect the occurrences and abundance of three *Silene* species derived from **(A)** Random Forest (RF); and **(B)** Canonical Correspondence Analysis (CCA). Eigenvalues for CCA1 and CCA2 are 31.46% and 56.48%, respectively. OM, organic matter; and EC, electric conductivity.

For three *Silene* species, Random Forest (RF) predicted the most important habitat variables (**Figure 3A**). Elevation, clay fraction, silt fraction, moisture content, sand fraction, and slope are the most important variables with high values of Mean Decrease Gini values (>1.0). In addition, all other variables highly contribute to the habitat quality of *Silene* species with Mean Decrease Gini importance values ≥0.33.

In addition, the Canonical Correspondence Analysis (CCA) elucidated the correlation between the abundance of Silene species and measured variables (**Figure 3B**). *S. leucophylla* demonstrated a strong correlation with habitat-type, sand fraction, and moisture content, whereas *S. schimperiana* showed positive correlations with chlorides, electric conductivity, slope, organic matter and clay fraction. On the other hand, *S. oreosinaica* exhibited positive correlations with elevation, silt fraction, and magnesium, and negative correlations with slope and habitat type.

On the other hand, the habitat quality variables of three *Silene* species are displayed in **Supplementary Table S3**. Except for sand fraction, pH, electric conductivity, SO_4_, Ca^2+^, aspect, slope, and distance to the nearest track, the other measured variables were significantly diverse at p≤ 0.05. The habitat of *S. leucophylla* had the highest values of moisture content (20.07%) and slope (30.71%), but the lowest values of CaCO_3_ (8.31%), Mg^+2^ (4.96 mEq/L) and elevation (1826 m). *S. schimperiana* habitat recorded the highest mean values of clay fraction (14.67%), organic matter (4.62%), Cl^-^ (14.27 mEq/L), habitat-type (2.0), and threat-type (1.70). Additionally, the habitat of *S. oreosinaica* attained the highest mean values of silt fraction (5.32%), CaCO_3_ (18.47%), K^+^ (6.87 mEq/L), Mg^+2^ (9.16 mEq/L) and elevation (2184 m).

### 3.3 Predictive current distribution, model performance, and key environmental predictors

**Figure 4:**
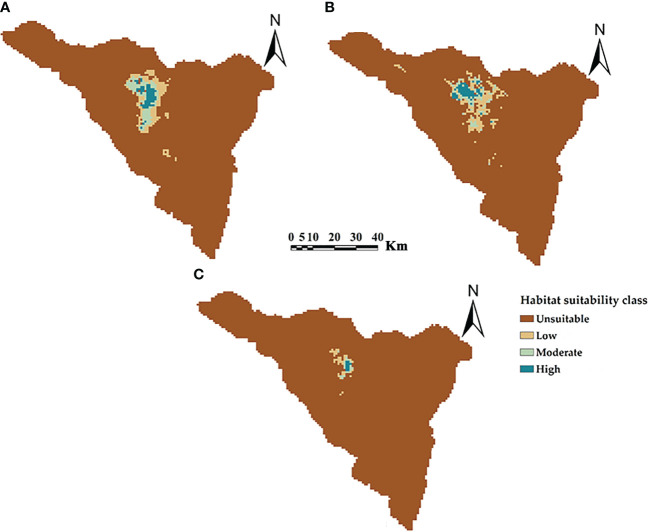
The predictive maps for the current potential distribution of three *Silene* species: **(A)** *S. leucophylla*; **(B)** *S. schimperiana*; and **(C)** *S. oreosinaica* in St. Catherine Protectorate, Egypt. Maximum training sensitivity plus specificity (MTSS) threshold values are 0.20, 0.16 and 0.30 for *S. leucophylla*, *S. schimperiana*, and *S. oreosinaica*, respectively.

The potential current distributions and suitability classes for three *Silene* species in St. Catherine, Egypt are shown in **Figure 4**. The middle northern sector of St. Catherine is identified as the hotspot location for the distribution of three *Silene* species with high potential suitable areas. According to the potentially suitable areas, *S. schimperiana* had the largest areas (293.10 km^2^, 5.64% of St. Catherine’s total area), followed by *S. leucophylla* (291.40 km^2^), while *S. oreosinaica* attained the lowest suitable areas (56.63 km^2^).

**Figure 5:**
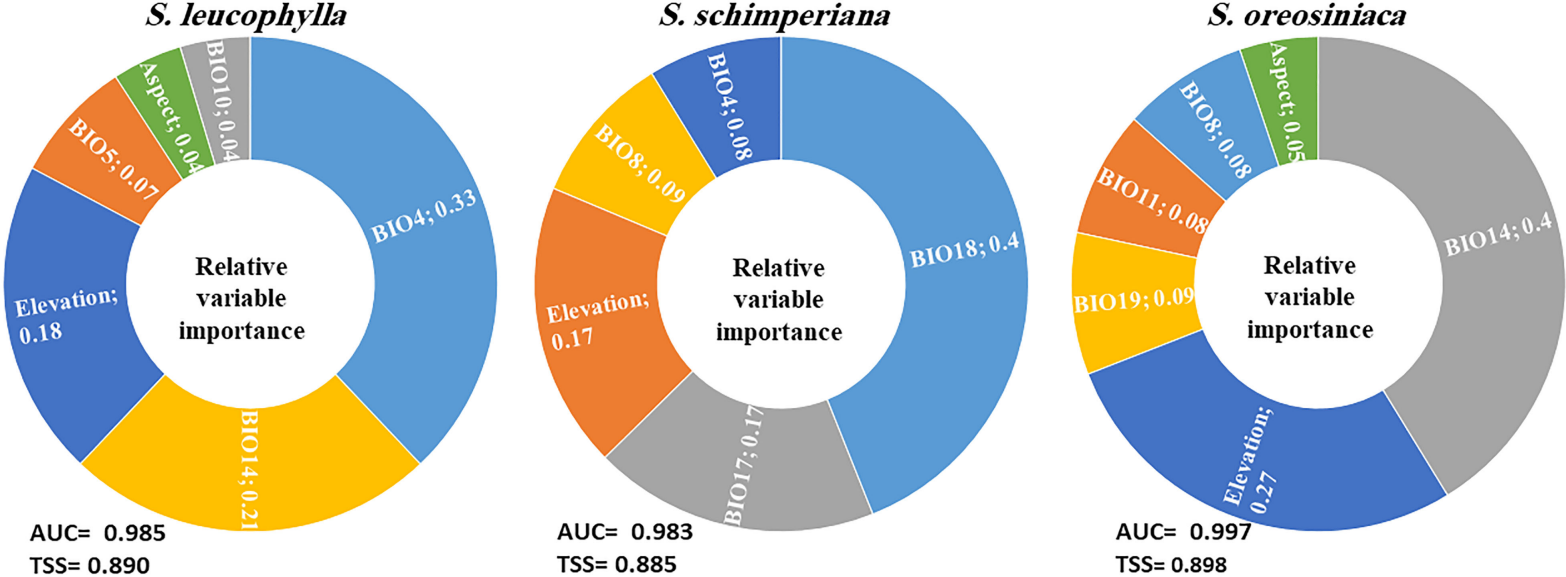
The relative importance of the environmental predictors explaining the potential distribution of three *Silene* species. The averages of the AUC and TSS indicate the accuracy of the ensemble models. BIO4, temperature seasonality; BIO14, precipitation of the driest month; BIO5, maximum temperature of the warmest month; BIO18, precipitation of the warmest quarter; BIO17, precipitation of the driest quarter; BIO8, mean temperature of the wettest quarter; BIO19, precipitation of the coldest quarter; and BIO11, mean temperature of the coldest quarter.

The average AUC and TSS values of single models and ensemble models for three *Silene* species were calculated (**Supplementary Table S4**). As compared with the three single models (GLM, RF and BRT), the ensemble models for three *Silene* species achieved higher values of AUC and TSS, indicating the high accuracy of ensemble models. The ensemble models’ AUC for *S. leucophylla, S. schimperiana,* and *S. oreosinaica* were 0.985, 0.983, and 0.997, respectively (**Figure 5**). These values revealed excellent model performance for three *Silene* species. A similar pattern of TSS values (0.890 for *S. leucophylla*, 0.885 for *S. schimperiana*, and 0.898 for *S. oreosinaica*) was obtained, confirming the reliability of the predictive models.

Concerning relative variables importance, the most important environmental variables for *S. leucophylla* were temperature seasonality (BIO4), precipitation of the driest month (BIO14), and elevation (**Figure 5**). For *S. schimperiana*, precipitation of the warmest quarter (BIO18), precipitation of the driest quarter (BIO17), and elevation were the main variables with a total importance of 74%. Among the selected variables, precipitation of the driest month (BIO14), elevation, and precipitation of the coldest quarter (BIO19) were the most imperative variables affecting the potential distribution of *S. oreosinaica* (**Figure 5**).

### 3.4 Predictive distribution range change under climate change

**Figure 6:**
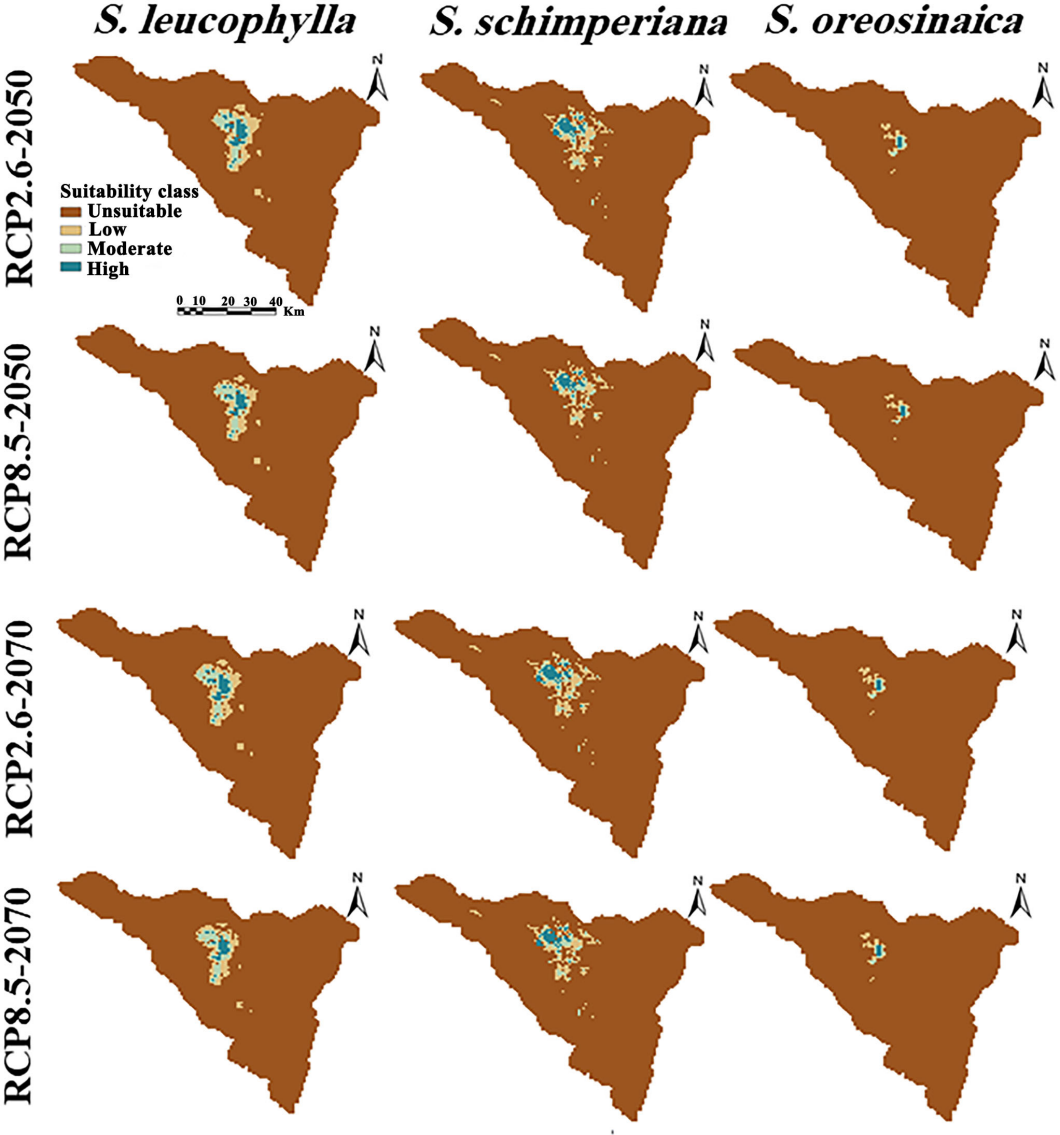
Potential future distributions of three *Silene* species under the optimistic low (RCP2.6) and pessimistic high (RCP8.5) emission scenarios. Columns from left to right show the projections of *S. leucophylla*, *S. schimperiana*, and *S. oreosinaica*, respectively.

By subtracting the predicted current distribution (**Figure 4**) from the predicted future distribution (**Figure 6**) yields the change in the distribution range of three *Silene* species (**Table 2**). The predicted future distribution of three *Silene* species in all climate scenarios (RCP2.6 and RCP8.5) over time (2050 and 2070) showed a progressive loss in suitable areas than the predicted current distribution.

**Table 2:** The estimation of fluctuations in the potential distribution of three *Silene* species under two future climate change scenarios.

Species	Suitable area change	Current	Future scenario			
			2050		2070	
			RCP2.6	RCP8.5	RCP2.6	RCP8.5
***S. leucophylla***	Suitable area (km^2^)	291.40	280.60	255.60	268.10	243.10
	Changed area (km^2^)	–	-10.80	-35.80	-23.50	-48.30
	Percentage of change (%)	–	3.70	12.28	8.06	16.57
***S. schimperiana***	Suitable area (km^2^)	293.10	274.75	243.94	265.60	241.48
	Changed area (km^2^)	–	-18.35	-49.16	-27.49	-51.62
	Percentage of change (%)	–	6.26	16.77	9.38	17.61
***S. oreosinaica***	Suitable area (km^2^)	58.29	55.75	53.25	55.40	46.60
	Changed area (km^2^)	–	-2.54	-5.04	-2.89	-11.69
	Percentage of change (%)	–	4.36	8.65	4.96%	20.05

Under RCP2.6–2050, RCP2.6–2070, RCP8.5–2050, and RCP8.5–2070 scenarios, the current suitable area of *S. leucophylla* was projected to decrease by 3.70%, 8.06%, 12.28%, and 16.57%, respectively. By 2050 and 2070, under the two RCP2.6 and RCP8.5 scenarios, the potential current suitable areas for *S. schimperiana* would decrease by 6.26%, 16.77%, 9.38%, and 17.61%, respectively. A comparable configuration was also established for *S. oreosinaica*, where the suitable areas would decrease by 4.36%, 4.96%, 8.65%, and 20.05%, respectively, under RCP2.6–2050, RCP2.6–2070, RCP8.5–2050, and RCP8.5–2070 scenarios (**Table 2**).

For three *Silene* species, no range expansion or gain in suitable areas was observed in the future under two climate change scenarios (RCP2.6 and RCP8.5) of 2050 and 2070. However, there is a gradually shrinking range in the north-central parts of St. Catherine with an upward shift to future climatically high-elevation areas. According to extinction risk, the studied *Silene* species can be ranked *S. oreosinaica*, then *S. schimperiana*, and finally *S. leucophylla*.

## 4 Discussion

By comparing current field observations with previous historical data, there is a continuous decline in the total number of individuals, with few mature individuals among the three studied *Silene* species. Moreover, based on current distribution modelling, the distribution ranges of three *Silene* species are highly fragmented due to mountain barriers, which are common in the study area (Zahran and Willis, 2009). Owing to their feeding properties as grazing plants, and the ruthless long-term drought, the populations of three *Silene* species are declining. Due to overgrazing and overcollection, the flowering and fruiting organs are eradicated, so the plants lose the ability to regenerate and hamper their life cycle. Moreover, the narrow distribution ranges at high elevations in rocky crevices with small and highly fragmented populations may also impede the expansion of their range and dispersal. Therefore, due to range restrictions, continuous human threats, and predicted climate change, the studied *Silene* species are vulnerable to extinction risk.

Both RF and CCA displayed the importance of elevation, soil texture (silt, sand and clay fractions), soil moisture, habitat-type, chlorides, electric conductivity and slope as among the main predictors affecting the habitat preferences and distribution of three *Silene* species in St. Catherine, Egypt. The three *Silene* species preferred rocky slope soil with a sandy texture, alkaline, and slightly saline soil. The significant variation in some soil features (silt fraction, clay, moisture, organic matter, calcium carbonates, and chlorides) among the three *Silene* species may be attributed to topography and plant composition (Shaltout et al., 2015). Many ecological factors and microhabitats support the existence of endemic plants within St. Catherine (Khedr, 2021). For example, rocky ground such as granite, cliffs, eroded soil, and a higher altitude range of mountains, as well as a unique geological structure, made St. Catherine an important area of endemism in Egypt (Moustafa and Klopatek, 1995; Danin, 2008; Hosni et al., 2013; Abdelaal et al., 2018).

In general, the St. Catherine mountain species are susceptible to temperature, and rainfall changes (Moustafa and Klopatek, 1995; Grainger and Gilbert, 2008; Abdelaal et al., 2020a). Moreover, some studies have emphasized the significance of microhabitat specificity for endemic species of the South Sinai (Ayyad et al., 2000; Khedr, 2007; Shaltout et al., 2015, Shaltout et al., 2016). These microhabitats are constructed by elevation, slope degree, and aspect direction. Our field observation showed that *S. leucophylla* and *S. schimperiana* share the same microhabitats, where they were recorded in slopes, gorges, and terraces in all aspects except flat and south-facing sites, while *S. oreosinaica* was only recorded in a slope microhabitat in the north direction. This finding was also confirmed by previous studies (Fakhry et al., 2019; Rabei et al., 2021). Thus, these species might receive little solar radiation, low temperatures, and high moisture as compared with steep south-facing sites where hotter and drier conditions prevail (Khedr, 2007; Abdelaal et al., 2020a).

For current climate conditions, the three *Silene* species inhabit the higher-elevation areas within the central northern sector of St. Catherine. These findings were consistent with our field observations and historical distributions reported in the literature (Moustafa et al., 2001; Zahran et al., 2015; Abdelaal et al., 2018; Fakhry et al., 2019; Rabei et al., 2021). Both *S. leucophylla* and *S. schimperiana* have wide distribution ranges as compared with *S. oreosinaica.*

On the other hand, the relative importance of the investigated bioclimatic and topographic predictors varied among *Silene* species. In addition to elevation, the predicted current distribution of *S. leucophylla* was more sensitive to temperature seasonality, and precipitation of the driest month, however, precipitation-related variables (BIO14, BIO17, and BIO18) were among the important variables controlling the current distribution of both *S. schimperiana* and *S. oreosinaica.* This indicates that the three *Silene* species could be more vulnerable to global warming (i.e. an increase in temperature and a decrease in precipitation) than other common mountain species in the study area. However, evidence is required to support whether the studied *Silene* species are physiologically able to resist some degree of hot and dry conditions. These findings are in accordance with other studies that applied SDM to address the potential geographical distributions for rare, endemic, and medicinal plant species in similar and related environments such as *Daphne mucronata* in Central Iran (Abolmaali et al., 2018), *Nepeta binaloudensis* in Khorassan–Kopet Dagh (Erfanian et al., 2021), *Pomatosace filicula* in the Qinghai–Tibet Plateau (Chen et al., 2022) and *Allium* species in Iran (Nasab et al., 2022), where they reported the vital role of climatic factors and elevation in shaping the plants’ distribution. A similar finding was also reported for endemic plants in the Kashmir Himalaya (Mir et al., 2020), which underlined the importance of temperature seasonality (BIO4), precipitation of the driest month (BIO14), and elevation. Furthermore, in Egypt, the spatial distributions of medicinal plants (Kaky and Gilbert, 2016, Kaky and Gilbert, 2017; Kaky et al., 2020) and endemic plants (e.g. *Rosa arabica* and *Primula boveana*) (Abdelaal et al., 2019; Abdelaal et al., 2020a) were closely controlled by precipitation and temperature-related variables and elevation.

The predicted current suitable distribution areas for three *Silene* species were characterized by moist conditions, low temperatures, and high elevation ranges (≥1600 m for *S. leucophylla*, ≥1550 for *S. schimperiana*, and ≥2000 m for *S. oreosinaica*) (**Figure 4**). These findings are in line with several studies (Moustafa and Klopatek, 1995; Moustafa et al., 2001; Zahran et al., 2015; Abdelaal et al., 2020a) that documented that, most endemic species of St. Catherine prefer moist microhabitats in rock crevices at high elevations with cold temperatures and moderate rainfall. Moreover, the models’ results for the current potential distribution of three *Silene* species exhibited some climatically topographically suitable sites within St. Catherine, despite our field surveys and literature that did not indicate the occurrence of these species in this mountain range. This may be due to three hypotheses: firstly, the possible extinction or disappearance of these species in those suitable sites (Rabei et al., 2021). Second, due to poor dispersal, the endemic species did not occupy all the available space and have not been recorded before in these suitable sites and conditions, or finally, a lack of adequate field surveys, especially for high-elevation rugged mountain areas. A similar concern has been addressed for *Rosa arabica* and *Primula boveana* in the same study area (Abdelaal et al., 2019; Abdelaal et al., 2020a) and *Nepeta binaloudensis* in Iran (Erfanian et al., 2021). However, additional floristic surveys are encouraged at these sites to look for new *Silene* populations or to find out what circumstances have made it difficult for these species to recruit or colonize all suitable areas.

In response to climate change, some species might benefit through range expansion and acquiring additional suitable areas for their survival and persistence, while others are negatively impacted by range contraction or habitat loss (Thuiller et al., 2005; Sintayehu, 2018). The three *Silene* species showed range-shift by moving upward to high-elevation areas in response to the increasing severity of climate change scenarios from RCP2.6 up to RCP8.5. However, this upward range shift was accompanied by a gradual contraction in their geographical range. Montane plant species that populate high-elevation areas are more susceptible to range shifts or extinction threats owing to climate warming (Lenoir et al., 2008; Dullinger et al., 2012; Dakhil et al., 2021). A similar pattern was also reported for the two endemic species, *Rosa arabica* and *Primula boveana* (Abdelaal et al., 2019; Abdelaal et al., 2020a). Range contraction was also predicted for several species in mountain areas and related arid environments, for example, *Nepeta binaloudensis* in Iran (Erfanian et al., 2021), *Tilia* species in Turkey (Canturk and Kulaç, 2021), and *Horsfieldia tetratepala* in China (Cai et al., 2022). Range-restricted and mountain-top species respond differently to climate change, either through range contraction or shifting their range to higher elevations. A study investigating climate change influences on the flora of the South Sinai documented a considerable upslope shift in its elevation-limits (Coals et al., 2018). As a result, it is predicted that, global warming could have a negative effect on narrow-range and endemic plants in St. Catherine, where mesic microhabitats and high altitudes exist.

Despite the St. Catherine is home to 14 exclusive endemic vascular plants and is accounted as the most floristically rich region in Egypt, coupled with the availability of laws that forbid the illegal collection, exploitation, and overgrazing of these species, their strict implementation is negligible. Threats such as narrow-range distribution, habitat specificity, drought, and overgrazing, all contribute to the ongoing population decline of three *Silene* species. Therefore, the studied *Silene* species are threatened and subsequently endangered according to the International Union for Conservation of Nature (IUCN, 2023). Thus, a sustainable conservation agenda is required to prevent the further loss of these species.

In conclusion, this study reported the continuous decline in the population size of *S. leucophylla, S. schimperiana,* and *S. oreosinaica*. Furthermore, the potential geographic distribution of three *Silene* species might undergo upward range shifts in response to climate change. Therefore, as high-altitude sensitive plants, the three *Silene* species may not be able to withstand future global warming. In order to reduce the risk of extinction, a thorough survey, ex-situ conservation, and translocation actions with regular monitoring of species are needed. The potential suitable areas under both current and future climates could be considered a decision-support tool for conservation planning and restoration strategies for the three *Silene* species. Besides, fenced enclosures and tissue culture propagation, followed by regular monitoring, are required after reintroduction into the proposed refugia. These actions should be supported by increased public awareness and policy initiatives to mitigate the effects of human activity. Finally, our methods could be replicated for other endemic and endangered species all over the world and can be done by conservationists, ecologists or relevant researchers. Regarding the conservation interest, our study highlighted the most important environmental variables and predicted suitable sites that should be considered during the reintroduction, or in-situ conservation of three *Silene* species in response to climate change, as well as giving an alarm to the rate of population decline of the three *Silene* species.

## Data availability statement

The original contributions presented in the study are included in the article/**Supplementary Material**. Further inquiries can be directed to the corresponding author.

## Author contributions

MA: Conceptualization, Data curation, Formal analysis, Investigation, Methodology, Software, Writing – original draft, Writing – review & editing. AA-H: Formal analysis, Funding acquisition, Investigation, Methodology, Project administration, Writing – review & editing, Conceptualization, Writing – original draft. SA: Investigation, Validation, Visualization, Writing – review & editing, Supervision. HA-H: Investigation, Validation, Visualization, Writing – review & editing. SA-R: Investigation, Validation, Visualization, Writing – review & editing. AA: Data curation, Investigation, Validation, Visualization, Writing – review & editing. MD: Data curation, Validation, Writing – review & editing, Formal analysis. RE-B: Data curation, Validation, Writing – review & editing, Formal analysis. AY: Data curation, Validation, Visualization, Writing – review & editing, Formal analysis.
