# Supplementary material for: Population status, habitat preferences and predictive current and future distributions of three endangered Silene species under changing climate
Source: Front Plant Sci. 2024 Jun 20;15:1336911. doi: 10.3389/fpls.2024.1336911 (PMC11222647; doi:10.3389/fpls.2024.1336911)
Supplement: Supplementary file 1 [file Table_1.docx]

**Table S1.** Source and Variance Inflation Factors (VIF) of the environmental variables.

| **Type** | **Code/** **Unit** | **Environmental variable** | **Source and resolution** | **VIF** |
| --- | --- | --- | --- | --- |
| Bioclimatic variables | **Temperature-related variables** | | WorldClim v.2.1 (~1 km^2^) |  |
|  | BIO1 (℃) | Annual mean temperature |  | 8.90 |
|  | BIO2 (℃) | Mean diurnal range (max. temp–min. temp) |  | 12.10 |
|  | BIO3 (℃) | Isothermality (BIO2/BIO7) × 100 |  | 13.15 |
|  | BIO4 | Temperature seasonality (SD × 100) |  | 4.88 |
|  | BIO5 (℃) | Max temperature of the warmest month |  | 3.15 |
|  | BIO6 (℃) | Min temperature of the coldest month |  | 12.45 |
|  | BIO7 (℃) | Temperature annual range (BIO5-BIO6) |  | 8.10 |
|  | BIO8 (℃) | Mean temperature of wettest quarter |  | 4.05 |
|  | BIO9 (℃) | Mean temperature of driest quarter |  | 8.19 |
|  | BIO10 (℃) | Mean temperature of warmest quarter |  | 4.15 |
|  | BIO11 (℃) | Mean temperature of coldest quarter |  | 4.34 |
|  | **Precipitation-related variables** | |  |  |
|  | BIO12 (mm) | Annual precipitation |  | 10.90 |
|  | BIO13 (mm) | Precipitation of the wettest month |  | 13.12 |
|  | BIO14 (mm) | Precipitation of the driest month |  | 3.53 |
|  | BIO15 | Precipitation seasonality |  | 4.00 |
|  | BIO16 (mm) | Precipitation of the wettest quarter |  | 9.10 |
|  | BIO17 (mm) | Precipitation of the driest quarter |  | 2.59 |
|  | BIO18 (mm) | Precipitation of the warmest quarter |  | 4.85 |
|  | BIO19 (mm) | Precipitation of the coldest quarter |  | 2.76 |
| Topographic variables | Elevation (m. a.s.l) | Elevation | EarthEnv (1 km^2^) | 4.10 |
|  | Slope (%) | Slope |  | 10.68 |
|  | Aspect (degree) | Aspect |  | 4.98 |
