# Supplementary material for: Population status, habitat preferences and predictive current and future distributions of three endangered Silene species under changing climate
Source: Front Plant Sci. 2024 Jun 20;15:1336911. doi: 10.3389/fpls.2024.1336911 (PMC11222647; doi:10.3389/fpls.2024.1336911)
Supplement: Supplementary file 2 [file Table_2.docx]

**Table S2**. Population and microhabitat features of three *Silene* species in St. Catherine Protectorate, Egypt according to field observations (2021-2022).

| Feature | ***Silene leucophylla*** | ***Silene schimperiana*** | ***Silene oreosinaica*** |
| --- | --- | --- | --- |
| **Population features** | | | |
| Population size | 1000 | 900 | 60 |
| Mature individuals | 69 | 51 | 28 |
| Distribution within St. Catherine | Gebal Mousa, Shaq Mousa, El-Faraa, Wadi Gebal, Elgragenia, Shaq Abo-Hamman, Gebel El-Ahmar and Gebal Catherine | Shaq Mousa, El-Faraa, Wadi Gebal, Elgragenia, Gebal El-Ahmar and Serbal | Shaq Mousa and Gebal Catherine |
| **Micro-habitat features** | | | |
| Type | slopes, gorges, and terraces | terraces, gorges, and slopes | slopes only |
| Surface | Rocky crevices | Rocky crevices | Rocky crevices |
| Quality status | Continuous decline and fragmented | Continuous decline and fragmented | Continuous decline and fragmented |
| Elevation range (m a.s.l.) | 1700-2200 | 1300-2300 | 2000-2300 |
| Aspect (direction) | All aspects except South (S) and flat | All aspects except South (S) and flat | North only (North, Northeast and Northwest) |
| Commonly associated species | *Galium setaceum* Lam., *Pterocephalus* *sanctus* Decne., *Chiliadenus montanus* (Vahl) Brullo., *Echinops* *glaberrimus* DC., *Tanacetum sinaicum* (Fresen.) Delile ex K.Bremer & Humphries | *Alkanna orientalis* (L.) Boiss.*, Achillea fragrantissima* (Forssk.) Sch.Bip., *Anarrhinum* *forskaohlii* subsp. *pubescens* D.A.Sutton, *Echinops glaberrimus* DC., *Tanacetum sinaicum* (Fresen.) Delile ex K.Bremer & Humphries | *Scrophularia libanotica* Boiss. and *Tanacetum* *sinaicum* (Fresen.) Delile ex K.Bremer & Humphries |
| Threats | Overgrazing, drought, and overcollection | Overgrazing and drought | Overgrazing and drought |
| Fenced enclosure | None | Present (in Shaq Mousa) | None |
