# Supplementary material for: Population status, habitat preferences and predictive current and future distributions of three endangered Silene species under changing climate
Source: Front Plant Sci. 2024 Jun 20;15:1336911. doi: 10.3389/fpls.2024.1336911 (PMC11222647; doi:10.3389/fpls.2024.1336911)
Supplement: Supplementary file 3 [file Table_3.docx]

**Table S3.** Habitat quality variables (mean values± SE) for three *Silene* species.

| **Variable** | ***S. leucophylla*** | ***S. schimperiana*** | ***S. oreosinaica*** | ***P value*** |
| --- | --- | --- | --- | --- |
| **Soil features** | | | | |
| Sand (%) | 89.42±1.43 | 83.48±2.45 | 86.22±1.34 | 0.276 |
| Silt (%) | 3.38±0.69 | 1.85±0.22 | 5.32±0.83 | **0.006*** |
| Clay (%) | 7.20±1.01 | 14.67±2.31 | 8.43±0.70 | **0.010*** |
| Moisture content (%) | 20.07±3.52 | 7.62±3.43 | 7.67±2.01 | **0.034*** |
| pH | 7.91±0.15 | 7.89±0.23 | 7.99±0.12 | 0.116 |
| Electric conductivity (EC, µs/cm) | 753.32±176.31 | 881.90±208.87 | 410±112.32 | 0.141 |
| Organic matter (OM, %) | 4.05±1.38 | 4.62±1.11 | 2.38±0.43 | **0.014*** |
| CaCO_3_ (%) | 8.31±0.76 | 8.62±0.89 | 18.47±3.91 | **0.000*** |
| Cl^-^ (mEq/L) | 8.73±3.33 | 14.27±5.12 | 3.95±1.05 | **0.007*** |
| SO_4_ (mEq/L) | 23.23±2.26 | 6.43±1.98 | 12.48±2.91 | 0.075 |
| K^+^ (mEq/L) | 5.83±2.16 | 1.91±0.51 | 6.87±1.93 | **0.033*** |
| Ca^++^ (mEq/L) | 14.09±5.51 | 7.47±1.15 | 16.82±2.58 | 0.057 |
| Mg^++^ (mEq/L) | 4.96±0.53 | 5.38±0.41 | 9.16±1.42 | **0.000*** |
| **Topographic variables** | | | | |
| Elevation (m) | 1826±85.75 | 1882±102.10 | 2184±32.48 | **0.050*** |
| Aspect (°) | 94.02±36.61 | 139.17±26.74 | 144.49±43.89 | 0.223 |
| Slope (%) | 9.39±1.10 | 7.18±0.76 | 9.15±0.82 | 0.198 |
| Habitat type | 1.64±0.22 | 2.0±0.25 | 1±0.0 | **0.000*** |
| **Threats** | | | | |
| Distance to the nearest track (m) | 592.86±174.81 | 924.5±201.58 | 932.73±152.96 | 0.662 |
| Threat type | 1.35±0.13 | 1.70±0.15 | 1.09±0.09 | **0.002*** |

- Habitat type is ranked as 1: slopes, 2: gorges, and 3: terraces and averaged.

- Threat type is ranked as 1: drought, 2: over-grazing, and 3: over-collection, and averaged.

* Refers to mean values that are significantly different at *p*≤ 0.05.
