# Supplementary material for: Population status, habitat preferences and predictive current and future distributions of three endangered Silene species under changing climate
Source: Front Plant Sci. 2024 Jun 20;15:1336911. doi: 10.3389/fpls.2024.1336911 (PMC11222647; doi:10.3389/fpls.2024.1336911)
Supplement: Supplementary file 4 [file Table_4.docx]

**Table S4.** Evaluation metrics (mean values of AUC and TSS± SD) of the single models and ensemble models.

| **Model** | ***S. leucophylla*** | | ***S. schimperiana*** | | ***S. oreosinaica*** | |
| --- | --- | --- | --- | --- | --- | --- |
|  | **AUC** | **TSS** | **AUC** | **TSS** | **AUC** | **TSS** |
| GLM | 0.872±0.010 | 0.752±0.010 | 0.898±0.005 | 0.747±0.006 | 0.910±0.020 | 0.795±0.050 |
| RF | 0.912±0.004 | 0.790±0.005 | 0.925±0.004 | 0.789±0.010 | 0.930±0.050 | 0.762±0.006 |
| BRT | 0.890±0.001 | 0.728±0.005 | 0.886±0.006 | 0.772±0.001 | 0.920±0.025 | 0.729±0.040 |
| Ensemble | 0.985±0.006 | 0.890±0.000 | 0.983±0.060 | 0.885±0.005 | 0.997±0.010 | 0.898±0.020 |
